# Merging existing practices with new ones: the adjustment of organizational routines to using cancer patient pathways in primary healthcare

**DOI:** 10.1186/s12913-021-07348-6

**Published:** 2022-01-02

**Authors:** Petter Fjällström, Anna-Britt Coe, Mikael Lilja, Senada Hajdarevic

**Affiliations:** 1 Department of Nursing, Umeå University, SE-901 87 Umeå, Sweden; 2 Department of Sociology, Umeå University, SE-901 87 Umeå, Sweden; 3 Department of Public Health and Clinical Medicine, Unit of Research, Education, and Development, Östersund Hospital, Umeå University, SE-901 87 Umeå, Sweden; 4 Department of Public Health and Clinical Medicine, Family Medicine, Umeå University, SE-901 87 Umeå, Sweden

**Keywords:** Standardized patient pathways, Cancer, Organizations, Routines, Unintended consequences, Adaption, Primary care physicians, Primary care nurses, Grounded Theory Method, Group interviews

## Abstract

**Background:**

The introduction of new tools can bring unintended consequences for organizational routines. Cancer Patient Pathways (CPP) were introduced into the Swedish healthcare system in 2015 to shorten time to diagnosis and treatment. Primary healthcare (PHC) plays a central role since cancer diagnosis often begins in PHC units. Our study aimed to understand how PHC units adjusted organizational routines to utilizing CPPs.

**Method:**

Six PHC units of varied size from both urban and rural areas in northern Sweden were included. Grounded theory method was used to collect and analyse group interviews at each unit. Nine group interviews with nurses and physicians, for a total of 41 participants, were performed between March and November 2019. The interviews focused on CPPs as tools, the PHC units’ routines and providers’ experiences with using CPPs in their daily work.

**Results:**

Our analysis captured how PHC units adjusted organizational routines to utilizing CPPs by fusing existing practices with new practices to offer better quality of care. Specifically, three overarching organizational routines within the PHC units were identified. First, *Manoeuvring diverse patient needs with easier patient flow*, the PHC units handled the diverse needs of the population while simultaneously drawing upon CPPs to ease the patient flow within the healthcare system. Second, *(Dis) integrating internal know-how*, the PHC units drew upon internal competence even when PHC know-how was not taken into account by those driving the CPP initiative. Third, *Coping with unequal relationships toward secondary care*, the PHC units dealt with being in an unequal position while adopting CPPs instead further decreased possibilities to influence decision-making between care-levels.

**Conclusion:**

Adopting CPPs as a tool within PHC units brought various unintended consequences in organizational routines. Our study from northern Sweden illustrates that the PHC know-how needs to be integrated into the healthcare system to improve the use of new tools as CPP. Further, the relationships between different levels of care should be taken in account when introducing new tools for healthcare. Also, when adopting innovations, unintended consequences need to be further explored empirically in diverse healthcare contexts internationally in order to generate deeper knowledge in the research area.

## Background

In several countries, Cancer Patient Pathways (CPP) have been introduced to shorten the time to diagnosis and treatment in cancer care [1–5], constituting an innovation in complex healthcare systems [6]. This new tool was introduced in Sweden in 2015 [4], upon influence from Denmark [2, 7] and included both Primary HealthCare (PHC) and secondary healthcare [4, 8]. CPPs are a standardized tool used from the first suspicion of cancer through diagnosis until either start of treatment or when suspicion of cancer has been eliminated [4]. The first suspicion of cancer is detected through pre-specified symptoms referred to as alarm symptoms, and in Sweden, these are often detected by PHC. Although the intention with CPPs is to improve quality of care and speed up time to diagnosis and treatment, as with any innovation, it likely has unintended consequences as described by Merton [9] for the healthcare organizations that adopt them. This article focuses on the unintended consequences for organizational routines in PHC.

The Swedish healthcare system is publicly funded and has a decentralized structure where the national government is responsible for deciding national health policies. In turn, twenty-one county councils implement these national policies and provide healthcare services autonomously through hospitals and PHC units [4, 10]. PHC units are particularly affected by the adoption of CPPs since they have long been the main point of entrance for people seeking care and most patients with diagnosed cancer present their alarm symptoms in PHC units [11]. Nurses in Swedish PHC units are often patients first contact and schedule the physician’s appointments while also having their own patients. Thus, PHC units are important actors for early detection, timely diagnosis and referrals to secondary care for treatment. Before the introduction of CPP, physicians in PHC units used their clinical judgement to assess patient’s symptoms and determine if and which additional tests and consultations were needed, for example, X-ray and colonoscopy. Physicians in PHC units were responsible for the entire investigation process during which a patient underwent tests and consultations in multiple steps until the physician could make a clinical diagnosis or had sufficient suspicion of cancer. At that point, they could refer the patient to secondary care. Since the introduction of CPPs, physicians in PHC units are expected to look for specific alarm symptoms that are associated with different forms of cancer when assessing patients, and if found, start a CPP for that type of cancer. In alignment with the specific alarm symptoms found, CPPs allow the physician to refer a patient into a standardized investigation for that type of cancer without the patient returning to the physician in the PHC unit after each investigation step. To sum up, before the introduction of CPP, PHC units had guidelines but no standardized tool to use when they assessed and referred patients for possible cancer, while CPP constitutes a new tool that attempts to standardize the entire process (with the intention of accelerating it). Nonetheless, adopting a new tool can lead to unintended consequences [9] especially when it meets with the already existing organizational routines created for this purpose.

Unintended consequences are the changes brought by an intervention in an organization other than those it aims to achieve [12]. As a concept, unintended consequences have been widely used in other research areas, while sparsely used in empirical studies within healthcare organizations. However, previous studies show that new initiatives in healthcare can affect their services. For instance, a centrally led health reform in Kenya resulted in a lack of clarity regarding roles and responsibilities at different levels in organizations, contributing to confusion and causing interruptions such as lack of drug supply delivery [13]. Moreover, healthcare providers can be affected. For example, the adoption of an electronic health record system in Scotland caused uncertainty among providers about how the patient story should be recorded and further communicated in the organization [14]. Similarly, in the U.S. providers increased their use of paper notes when lacking sufficient cognitive support from the adopted health record system [15]. Unintended outcomes are not necessarily negative. Pomey et al. [16] observed positive outcomes in operational enhancement in other parts of the healthcare system when strategies were introduced to improve access to surgery.

Unintended consequences can also be triggered by other organizational factors apart from the intended plan of a new initiative. Van de Ruit [17] found that structural and local factors, such as the absence of coordination and regulation, contributed to adverse patient outcomes when a global health policy program was adopted in South Africa in a setting with already low capacity. Previous research further showed factors such as insufficient preparedness and capacity for change decreased motivation in healthcare providers when adopting results-based financing in health systems in Zimbabwe [18]. Thus, conditions of the organizational environment as well as in the organization itself shape the adoption of a new initiative and hence the unintended consequence.

Organizational routines are the repetitive, recognizable pattern of interdependent actions that involve multiple actors [19]. In their execution through work practices, organizational routines are subject to continual change, and therefore can be better understood as “work in progress” rather than finished products [20]. Moreover, organizations practice similar routines in diverse ways due to different resources and capacities, geographical variances or lacking certain competencies [8, 21]. Routines fill different functions in any organization, and three main functions that have been conceptualized are coordination, (un)learning and truces [22–24]. In healthcare, organizational routines structure the daily work practices of healthcare professionals and administrators on all care levels and are key to maintaining and improving the quality of care [25]. Organizational routines are often the target of new initiatives but are also affected by them. Novak et al. [26] found unintended consequences for other intersecting organizational routines during the adoption of an IT-driven routine which led to earlier stable intersections of routines becoming misaligned. Research also shows how organizations adjust to the unintended consequences of new initiatives. For instance, adopting a new telehealth service disturbed existing ones and contributed to fragmentation, changed responsibilities and adjustments to balance standardized practice with local innovation [27].

We approach the PHC units in our study as organizations because they comprise both a production system dependent on resources, able to produce healthcare service and purposively constructed to achieve predefined goals; as well as a social system with individuals and social groups trying to adapt and affect relations in organizations [28]. Moreover, we understand CPPs as an innovation in these organizations since they represented a new tool that the PHC units are required to use.

Our study aimed to explore how the PHC units were adjusting their organizational routines to utilizing CPPs. Specifically, we wanted to understand the longstanding routines in the PHC units to assess, identify, and refer potential cancer cases to additional care as well as in what ways these longstanding routines were affected by the adoption of CPPs as a tool designed for these same purposes. Following the research outlined above, we found the concept of unintended consequences suitable for interpreting our study results. As we show below, the adoption of CPPs in these PHC units had unexpected consequences for the longstanding organizational routines to assess, identify and refer potential cancer cases.

## Method

Constructivist Grounded Theory Method [29] was used to collect and analyse qualitative data through group interviews with physicians and nurses in PHC units. This method allowed us to explore the meanings and actions assigned to everyday work in the units: routines developed to handle patients and their symptom presentations; the use of CPPs and how ways of working have been affected when adopting CPPs.

### Setting, recruitment and participants

The study was conducted in PHC units within the healthcare system in northern Sweden. We sought to include PHC units in urban and rural areas with a variation in unit sizes. Recruitment began with purposive sampling of PHC units and participants following our aim and inclusion criteria. Since we were finishing a previous study in PHC units, we first contacted three of these units and all accepted to participate. After analysing the data from these PHC units, we realized that more data was needed to develop our emerging categories and therefore decided to recruit three additional PHC units.

In each participating PHC unit, we included nurses and physicians as participants because they are the PHC providers whose daily work involves identifying alarm symptoms and utilizing CPPs. To recruit participants, we held a general informational meeting with nurses and physicians to explain the purpose of the study and how it would be carried out. Next, we sent a letter to managers in the separate PHC units to coordinate a meeting with nurses and physicians and a letter with specific information to them that outlined the terms of voluntary and informed consent to participate in the study; lastly, the group interviews were arranged at these PHC units.

### Data collection

Data were collected through group interviews in order to explore shared and contrasting views through interactions between participants as a group [30] and complemented with focus group techniques [31]. The number of participants in each group varied from two to eight depending on what was practically possible at the moment for each PHC unit. Characteristics of the PHC units, groups and participants are presented in Table 1. In the large and medium-sized PHC units, more participants had time to participate, therefore nurses and physicians were separated for discussion by profession groups (three groups each); whereas in the smaller PHC units, group interviews were conducted with a mix of nurses and physicians together (three groups, though one only included nurses, no physician was present). The interviews were conducted between March and November 2019 with nine separate groups where in total 21 nurses (all women) and 20 physicians (50% women) with a variety of experience (50% had worked more than 5 years in PHC) participated. Two of the authors (ABC and SH) and one PhD candidate paired in different constellations, where two interviewers conducted each group interview, one acted as the moderator and the other wrote field notes and added supplementary probes when needed. The interviewers, one from nursing sciences with clinical experience (SH or PhD candidate) and one from social sciences (ABC) were always paired together during the data collection process with an insider and outsider perspective.

**Table 1:** Characteristics of the PHC units; their setting, groups, and participants

PHC Units	Areas	Listed patients	Group interviews^a^	Groups	Nurses	Physicians
Unit 1	Urban	10,000–15,000	Separate	2	8	8
Unit 2	Urban	15,000–20,000	Separate	2	5	3
Unit 3	Rural	< 5000	Mixed	1	2	5
Unit 4	Rural	< 5000	Nurses only	1	2	0
Unit 5	Urban	5000–10,000	Separate	2	3	3
Unit 6	Rural	< 5000	Mixed	1	1	1

^a^Group interviews were conducted with nurses and physicians in separated groups on different occasions or mixed groups depending on PHC unit size and what was possible for them

A semi-structured interview guide with open-ended questions was used, initially, questions addressed PHC providers’ routines directly related to the adoption of CPP. However, after the first interview with nurses, we realized that not all nurses were familiar with CPPs in a strict sense. Therefore, we broadened our questions to encompass routines regarding how providers understood and handled patients who described symptoms that could signal a serious illness such as cancer; how they channelled these patients to further care when needed; and how PHC providers understood their routines had changed or not over time. In subsequent interviews, participants’ answers became richer and they felt more comfortable discussing their work. Throughout data collection, we added new questions regarding emerging categories.

All interviews were conducted during participants working hours at their workplace and audio recorded. The interviews lasted from 37 min to 56 min (mean 46) and were verbatim transcribed and inserted into MAXQDA 2018, a software data program for coding and analysis.

### Data analysis

During data analysis, we followed the coding techniques of Grounded Theory method to perform constant comparison between empirical data, codes and memos [29]. Initial coding began as soon as the first transcription became available, and memos were written. Initial coding entailed analysing transcripts line-by-line to interact with each fragment of the data and label it. Doing this allowed us to determine the need for further data collection as initial codes pointed to emerging categories. After completing nine group interviews and initial coding of the entire data set, focused coding was conducted that entailed sorting and grouping the initial codes into categories. Continuing with constant comparison, the categories and the relationship between them were analysed and theorized. Concretely, we interpreted our categories as the work routines of the PHC unit and linked our interpretation to the concept of organizational routines. All authors have been involved throughout each analysis step contributing with both insider and outsider perspectives. Furthermore, within the analysis, we have been structured in method and discussed our preconceptions in each step to stay open and ground our analysis in the data, while at the same time flexible by using memos to spark ideas and identifying connections and patterns.

## Results

**Fig. 1:**
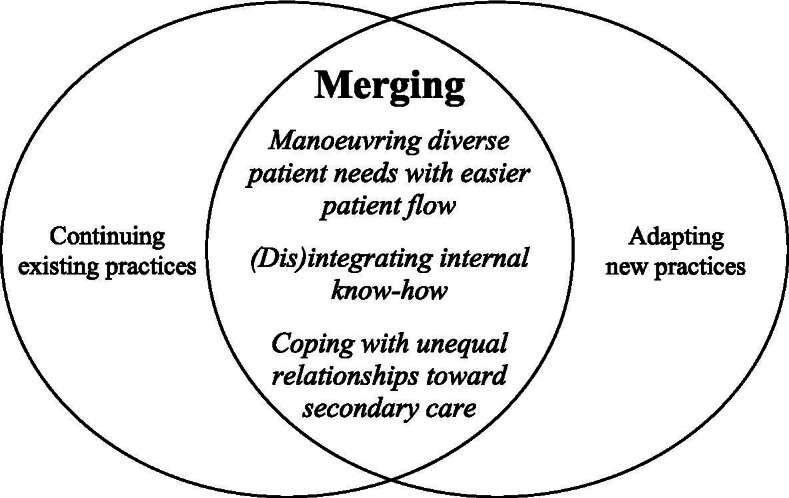
Fusing existing organizational routines with CPPs to offer better quality of primary healthcare

Our analysis captured how the PHC units had adjusted their organizational routines to utilizing CPPs by fusing already existing routines to detect suspected cancer cases with the CPPs for this same purpose. That is, rather than replacing already existing organizational routines with CPPs or displacing CPPs with already existing routines, the PHC units merged these together, as illustrated in Fig. 1. Specifically, we found three organizational routines had been adjusted: *Manoeuvring diverse patient needs with easier patient flow; (Dis)integrating internal know-how;* and *Coping with unequal relationships toward secondary care*. Each organizational routine encompassed dimensions of continuing existing practices and of adapting new practices by using CPPs. In Table 2, we present an overview of our categories and subcategories including the two dimensions. In the remainder of this section, we present the three central organizational routines. In the discussion, we integrate our results with the existing research on unintended consequences of innovations for healthcare organizations.

**Table 2:** Overview of categories and subcategories in dimensions

Dimensions through our sub-categories		Categories	
Continuing	Adapting	Organizational routines	
Having a broad approach to meet diverse needs of the population	Using CPPs to simplify work while being limited	Maneuvering diverse patient needs with easier patient flow	Merging existing practice with new ones
Managing the patient flow in and out of primary healthcare			
Knowing how to act as patients’ first point of contact with care	Using CPPs enhancing physicians’ know-how while mostly excluding nurses	(Dis)integrating internal know-how	
Drawing upon (accumulated) expertise of all profession groups			
Being reliant on secondary care	Using CPPs intensifying secondary care control instead of empowering primary healthcare	Coping with unequal relationships toward secondary care	
Experiencing one-way communication with secondary care			

### Manoeuvring diverse patient needs with easier patient flow

This organizational routine captured how the PHC units in our study handled the diverse needs of the population while simultaneously drawing upon CPPs to ease the patient flow by moving patients with suspected cancer more quickly through the healthcare system. This organizational routine consisted of two ways in which the PHC units continued to work as usual and one way in which they adapted to using CPPs.

The first way of continuing as usual consisted of the PHC units maintaining a broad approach to dealing with the diverse needs of a large number of patients to ensure that those who need healthcare receive it. Because the PHC units were typically the first point of contact for the population, nurses and physicians described that they frequently met patients with diverse symptoms, varying in degree of seriousness, and felt a responsibility to accurately assess these symptoms. They also pointed out that cancer was not the only serious disease they had to consider in order to identify those in need of cancer care. When nurses investigated patients need first, they preserved this broad approach to ensure adequate help and accurate access to healthcare based on the diversity of patients’ needs. Nurses and physicians also emphasized their responsibility to observe and assess potentially serious symptoms and conditions, which the following quote from a mixed group interview illustrates:

*It's one of the big challenges in some way here (in the PHC unit), being like a fishing net. In a way that’s effective for the entire population, otherwise it easily happens, that we only have time for those we take in and the rest are left out. So we have to evaluate based on everyone in some way.*

(Mixed group interview, unit 6)

The second way of continuing as usual consisted of the PHC units managing the total patient flow of all patients by moving them in and out of the PHC units, including the referrals to and from secondary care when needed. PHC providers continued with already existing routines to maintain control over the patient flow. As before, nurses assessed and prioritized a variety of needs and symptoms as either acute or not during phone conversations or drop-in visits to the PHC units, to keep the patient flow going in accordance with the available capacity of the PHC units. Nurses expressed how they usually were accustomed to working innovatively and flexibly when booking appointments for patients to accommodate those needing further medical assessment by physicians, as explained in the following quote.

*(In telephone) If you meet clear symptoms that something is… that this person is really ill, then they can come here very fast, and then maybe you schedule in a way that does not fit these rules that we have here on XX (PHC unit), then you have to skip those routines so that the patient can come quickly to us.*

(Group interview with nurses, unit 4)

As illustrated, nurses reported awareness of alarm symptoms and quickly scheduled patients presenting such symptoms. In contrast, physicians were less accustomed to focusing on alarm symptoms to determine further flow. Following their usual way of working, they made decisions several times during the day and only a small part of the symptoms pointed to a serious illness such as a cancer diagnosis. Physicians felt a responsibility to make an independent clinical medical decision before determining whether there is a need for referral to secondary care without creating obstacles in the patient flow.

In addition to continuing as usual, the PHC units in our study adapted to using CPP by relying on these to speed up and simplify their work while simultaneously feeling limited by their narrow criteria. PHC providers described that CPPs sped up and simplified their work through the tool’s focus on *clear* alarm symptoms for specific cancer. Both nurses and physicians viewed CPPs as a useful national strategy that had reduced waiting time for patients with suspected cancer and facilitated access to cancer care. This was especially relevant for patients experiencing diffuse symptoms for whom physicians now could send a referral to secondary care in a specific CPP. Thus, physicians used CPPs as a complementary tool in their decision of whether or not to contact secondary care. CPPs was perceived by physicians as a supportive, concrete tool that helped clarify information for patients regarding continued healthcare as described in this quote:

*But I feel that it (CPP) is a support when working, it’s so easy to use. It’s not always so easy to use a tool in primary healthcare. / But it has sort of helped us to set it up in our mind, to have it as an obvious routine in our head.*

(Group interview with physicians, unit 1)

Even if CPPs sped up and simplified their work, it came with limitations. Nurses and physicians perceived that CPPs had a narrow focus on specific alarm symptoms that overlooked the unique experience of each patient. Physicians felt especially limited by this in complicated cases, such as patients with several diseases, because the vague symptoms experienced by patients are not accounted for by CPPs. They perceived that complicated cases were not suitable for such standardized tools and risked being excluded from the fast track that CPPs offered. By prioritizing CPPs, physicians understood that waiting times were being prolonged for other patient groups also in need of acute healthcare resources. Thus, the main limitation of using CPPs was the isolated focus on standardized patient’s symptoms and the risk of sending too many referrals. In a mixed group interview, the need to balance that focus with the assessment of each patient’s personal experience of symptoms was discussed:

*We are not robots, because then you could just as easily have everyone fill in, pick out, do you have blood? Here you have it and then a computer sends a CPP referral. So there is a point that we physicians should assess them here (PHC unit) and based on all circumstances make some form of assessment / so maybe you should question the standardized cancer patient pathways or at least the name / We need an individual assessment for each patient… because there are different patients but we can pick out the same symptoms, but they are not the same and they have different diseases and maybe not everyone has cancer, so for that reason.*

(Mixed group interview, unit 3)

It was the physicians who used CPPs for referrals within PHC units and nurses were not involved in providing such referrals. However, both nurses and physicians perceived the risk of missing patients’ experience of unique symptoms when using fixed templates of standardized symptoms. Instead, they both pointed out their independent clinical decisions as important to keep the focus on the patient’s experienced symptoms as a base for assessment to ensure patient flow in the PHC unit and further CPP referral.

### (Dis)integrating internal know-how

The second organizational routine captured how the PHC units in our study drew upon internal competence to adopt CPPs even when their know-how was not taken into account by those driving the CPP initiative. This organizational routine comprised two ways in which PHC continued to work as usual and one way in which they adapted to using CPPs.

The first way of continuing as usual consisted of the PHC units relying upon their internal expertise to act as patients’ first point of contact even in the case of serious illness. PHC units in Sweden are expected to provide patients with substantial care before, or without having to, refer them to secondary or even emergency care. This meant that both nurses and physicians already had well-developed expertise tailored to be the first point of contact with whom patients present their health concerns and receive relevant care. This expertise was especially noticeable among the nurses because they described having long ago acquired the know-how to assess whether patients had alarm symptoms and thereby quickly identify whether patients might be presented with a serious illness, including possible cancer. Importantly, nurses described having worked with alarm symptoms routinely in their daily work for much longer than the introduction of CPPs. Moreover, they pointed out that this routine has not changed when adopting CPP, as explained in this quote.

*Well, you still have alarm symptoms, we had that before too / Red flags / yes, which makes you think about who should come here quickly. I think it has existed before as well / yes, that's the way one has always thought.*

(Group interview with nurses, Unit 1)

Working with alarm symptoms was used as a tool to prioritize patients and in the PHC units, it was mostly nurses who first assessed how acute the patient situation was.

The second way of continuing as usual consisted of the PHC units in our study being able to draw upon the accumulated internal expertise among profession groups. Nurses and physicians faced numerous responsibilities that they needed to handle in their daily work and there was no scheduled time for them to sit down and share thoughts together. To cope with this pressured situation, they described longstanding competencies that they had developed, such as knowing how to ask for help and support from each other when they needed it. Because of their high workload when managing patients’ various needs, nurses and physicians acknowledged the importance of knowing how to turn to internal cooperation between different profession groups, especially nurses as described in the following quote:

*It became easier (with team meetings), especially on the phone. You do not have to carry the entire burden yourself and make an assessment / Yes / You ask yourself, how should I schedule this patient, there is no time. Now everyone had to share the burden, so I think it became easier for us nurses. / Because then we could involve occupational therapist, physiotherapist, / Yes, and the assistant nurses.*

(Group interview with nurses, Unit 4)

In interprofessional meetings, nurses valued being able to and knowing how to support each other between profession groups. According to nurses and physicians, everyday collaboration was needed for joint learning and management of the daily patient flow, where the focus is on trusting each other, reciprocal sharing and receiving support. However, depending on the size of the PHC units, different ways of cooperation have been used. In bigger units, physicians described a planned process used with targeted groups or scheduled time for discussions between profession groups. In smaller units, nurses and physicians expressed that they learn about changes, mostly during daily informal meetings and when all profession groups were gathered together. Here we noted a difference, even though nurses and physicians acknowledged the importance of cooperation; physicians, especially in the large units, described taking and offering support from other physicians in their units and other units. In contrast, nurses described taking and offering support from various profession groups within the units.

Alongside continuing as usual, the PHC units in our study had adapted to using CPPs by physicians enhancing their know-how and creating new practices with this know-how. Nurses had mostly been excluded from acquiring new knowledge about CPPs and from actually using the CPPs. In contrast, because they were required to use CPPs, physicians gained new knowledge about CPPs as a tool and applied this knowledge to develop new practices in the PHC, often gradually, as described in this quote:

*I think the adoption of CPP, for my part has probably been adopted step by step and then gradually built on… and I do not feel that it was a starting point, if you think as a process, it has sneaked into our unit.*

(Group interview with physicians, Unit 4)

All PHC units had in different ways created new work tasks in conjunction with using CPPs: isolated rectoscopy tracks, skin and subacute clinics; customized CPPs information and lists of physicians for acute cases; as well as recurring meetings in teams or between nurse and physician. Physicians thought that these new work tasks were a good way to integrate CPPs into existing practices. However, even though nurses were willing to go along with these new tasks, they were rarely involved in the decisions to create them and understood CPPs as a tool most relevant for physicians. One exception was in a PHC unit where a physician had motivated the entire staff around the adoption of CPPs. In the remaining PHC units, even while physicians used CPPs; nurses, were very little or not at all familiar with CPPs even though they assess alarm symptoms that could indicate cancer daily. Nurses appeared to lack information as described in the following quote:

*Because I also felt when there was talk about it (CPP), but I thought it is mostly the physicians that are involved… Because we have not received any information of our own here at XX (PHC unit) for example, that we should work like this or so.*

(Group interview with nurses, Unit 4)

When the PHC units adopted CPPs, nurses for the most part seemed to have been excluded. Even though nurses are almost always the personnel category that has the first contact with patients and faces the initial burden of determining relatively quickly whether the symptoms presented are serious and potentially suspect of cancer. Thus, when adopting CPPs, the PHC units missed out on important know-how that nurses as a group had accumulated concerning alarm symptoms and initial patient assessments.

### Coping with unequal relationships with secondary care

This third organizational routine captured how the PHC units dealt with being in an unequal position to secondary care while adopting CPP failed to change this position and instead further increased difficulty for them to influence decision-making between care levels. This organizational routine consisted of two ways in which the PHC units continued to work as usual and additionally one way in which they tried to adapt to using CPPs.

The first way of continuing as usual consisted of the PHC units relying on secondary care upon which they were dependent within the care chain. Physicians described an ongoing reliance on secondary care not only for specialist knowledge but also because they are in a subordinate position that produces an uneven playing field. Dependence on secondary care for specialist knowledge was described in this way:

*In some of these CPPs, there is a very high proportion of patients with malignancy and in some there is a quite low proportion actually. At that moment you are in the hands of the specialists, or perhaps, the secondary care who have more detailed knowledge.*

(Group interview with physicians, Unit 1)

Physicians perceived that this dependency further contributed to secondary care having more influence in decision-making, allowing secondary care to be in control. Physicians described a relation to secondary care where the PHC unit on the one hand put trust in secondary care, and on the other hand, did not have the same possibility to influence decision-making on shared guidelines between care levels.

The second way of continuing as usual consisted of the PHC units experiencing a one-way communication with secondary care. This one-way communication with secondary care began before the adoption of CPPs and persisted afterward. Physicians described how new criteria were included in referrals without their involvement and how information from secondary care did not seem to reach the PHC units. Moreover, physicians pointed to the absence of feedback as well as accessible information from secondary care regarding referral criteria. Instead, physicians explained how they stumbled upon updated information by chance when looking for other information or when referral criteria suddenly changed. Physicians described attempting to change the one-way communication, while at the same time lacking the necessary channels for such improvement and reciprocal learning, as illustrated from a mixed group interview:

*We have made attempts to get feedback, regarding the remittance responses and there has been some lunch regarding CPP, but not at the level that may be needed, where you meet up. It is a problem in general in the communication between hospitals and primary healthcare, not just regarding the CPP.*

(Mixed group interview, Unit 3)

It was mostly physicians that described how the factor of a gap in communication with secondary care and inaccessible information affected their everyday work. Physicians observed that this gap contributed to more responsibility and tasks being transferred from secondary care to the PHC units without more resources and no explanation as to why. In addition, physicians pointed out that in the one-way communication with secondary care, they were unable to contribute to improved criteria and referral pathways.

However, the way in which the PHC units had adapted to using CPPs appeared to further intensify secondary care control because the PHC units became the provider of a task that was already decided by the secondary care. Physicians experienced this imbalance especially when the criteria for referrals regarding CPPs were changed by secondary care without informing the PHC units, which also undermined the boundaries of responsibility. This despite that physicians in the PHC units considered the criteria in CPPs on paper to be clear and were willing to discuss it with secondary care to improve ways of working. However, secondary care still determined the rules of CPPs between them and were the ones making the decisions, as explained in the following quote:

*This is an ongoing discussion in the XX (group of physicians representing PHC units) about CPPs and various CPP flows that we see do not work. It is above all this with… who is responsible for the task? But it's a lot about who does what. Well, we think it's pretty clear and so the clinic (at the hospital) says no! And then there is a small line that you interpret a little differently. The clinic says, this is what you (PHC units) are supposed to do and then they toss it over.*

(Group interview with physicians, Unit 5)

When PHC providers used CPPs, an intensified secondary care control within the uneven playing field exacerbated the PHC units’ subordinate position and the communication gap between the two levels of care. For their work to function more smoothly, physicians preferred less territorial thinking between PHC units and secondary care, and a clearer division of responsibilities regarding the referral criteria for CPP. They instead focused their attention on empowering their position and strived to improve relations in the care chain. Nurses and physicians pointed out that if there were improved relations between levels of care, the PHC units would be in a better position to jointly use CPPs with secondary care. They perceived that using CPPs had not contributed to more joint efforts between care levels, however, physicians welcomed the goal in CPPs to involve and clarify the position of the PHC units in the patient flow. Physicians described adapting to using CPPs mostly on their own and in coordination with other PHC units to empower their position between care levels. The consequence instead was that nurses and physicians in the PHC units used CPPs on their own and looked after themselves. This in turn led to lacking possibilities for the PHC units to influence improving the use of CPPs in their context, for example, by further developing criteria and pathways to secondary care.

## Discussion

Our results indicate that the PHC units adjusted to utilizing CPPs by fusing existing and new practices within three organizational routines. This suggests that the adoption of CPPs had both intended and unintended consequences for the PHC units’ routines [9, 12]. As intended, PHC providers used CPPs as a tool to facilitate a faster patient flow within the PHC units by referring to the alarm symptoms associated with specific forms of cancer in each CPP and following the procedures described in the CPP accordingly. Even so, PHC providers observed that using CPPs risked prolonging the waiting time for other patient groups, as previous studies found in secondary care as well [32, 33]. Nonetheless, the bulk of our results show that the adoption of CPPs had unintended consequences for the organizational routines in the PHC units.

According to our results, the PHC units relied mainly on their existing practices to create new ones on their own because they were not involved in the larger decision-making and planning processes for the adoption of CPPs. Instead, regional authorities developed these processes with the involvement of secondary care levels. They continued being stuck in an already existing uneven playing field even after adopting CPPs, especially in relation to secondary care. In addition to these external divisions, we found internal divisions within the PHC units. Nurses were not involved in the adoption of CPPs because only physicians were assigned the responsibility of using this new tool. These unintended consequences contributed to a guarding attitude of “we and them” instead of a “we solve it together” ambition within the organization. Best and Andrews [34] found that when developing a qualitative improvement initiative, involving staff, increasing support and education and adopting practical tools led to positive attitudes of staff as well as more consistent care, which in turn impacted the quality of care. In contrast, the PHC providers in our study felt excluded from the development of CPPs and had to depend on their know-how, develop new skills and create new tasks on CPPs by themselves. They acted on their own because of their motivation to improve organizational routines and adjust these to offer quality care to patients.

Each of the three organizational routines in our findings relate to the functions that routines have been theorized to play in organizations: coordination, (un)learning and truces.

The organizational routine, manoeuvring diverse patient needs with easier patient flow, aligns with the function of coordination. Routines have a crucial function of coordinating different actions within an organization, similar to the muscular movement coordinating the body as it walks [22]. Okhuysen and Bechky [35] states that routine is one of the five mechanisms of coordination that embody people that work collectively, with independent work and the work or goal is achieved. As a new tool, the adoption of CPPs unexpectedly enhanced other routines, as previous research shows [16]. Our findings suggest that the PHC units strengthened the coordination of original actions designed to detect cancer, such as identifying alarm symptoms and acting fast to facilitate and improve referrals. As Hung et al. [36] found in their study, PHC providers found it easier to adopt an intervention when it directly benefited them to ease the organizational environment of an already overstrained workforce.

The organizational routine, (dis)integrating internal know-how, corresponds to the function of (un)learning. Routines maintain organizational learning over time and offer a memory structure of “the way things are done here” [37]. Meanwhile, learning something new involves unlearning the old way. Fiol and Connor [23] describe unlearning of organizational routines through their model that starts with destabilization, followed by discarding the old routine and resulting in learning a new routine through experimentation. Previous research shows that new procedures in organizations cause uncertainty when healthcare providers adopt the procedure [14, 15]. In our study, this did not appear to be the case, physicians and nurses were clear about their responsibilities and internal know-how for managing symptoms. A possible reason could be the decentralized system in Sweden where PHC providers are used to making their own decisions and integrating innovations or not into their internal know-how, as our results show. A report from Denmark, with a similar decentralized system, showed variations in the implementation of CPPs in different regions and PHC physicians felt different degrees of uncertainty regarding their tasks depending on how CPPs had been introduced [38]. Similarly, in our results, PHC providers instead felt uncertainty regarding how to best optimize CPPs within their own PHC unit. Even though they were not involved in its planning and did not receive training and support in the use of CPPs, they had to use this new tool. This uncertainty left an opening for variation between the PHC units in the use of CPPs, including a recognition of its limitation that allowed providers to lean on their already existing organizational routines for the same purpose.

The organizational routine, coping with unequal relationships toward secondary care, relates to the function of truces. The concept of truces was developed within early theorizing of a negotiated order in healthcare [39]. Truces operate to balance conflicting goals and interests within organizations, and as Salvato and Rerup show [24], organizational routines help create truces between different actors. Even though the PHC units in our results used CPPs as intended to facilitate and speed up the patient flow to secondary levels, barriers between primary and secondary care remained from before. Indeed, the adoption of CPPs had the unintended consequence of deepening this unequal relation and demanding that the PHC units to cope with this deepened position of dependency and subordination. Even though the PHC units attempted to re-negotiate a new truce of who does what between care levels with the adoption of CPPs, secondary care did not appear to join in the re-negotiation of this truce. Atherton [40] found that when organizations distribute new knowledge, it is crucial to include different levels of existing knowledge in organizations, individual, local, available and common. Our study found that within the PHC units, individual and local knowledge, both formal and informal, were taken into account by providers, even if this did not occur beyond the PHC units.

Our overarching result is that the PHC units adjusted to utilizing CPPs by merging longstanding existing practices and new practices together in a dynamic tension, where the three organizational routines were under continual change rather than finished products. This aligns with Greenhalgh et al.’s [41] conclusions that sustainable change in the healthcare system embodies a tension between the persistence of past practice and the adaptation to a changing context. Feldman [20] similarly describes organizational routines as a “work in progress” and argues that the dynamic tension in routines can affect and trigger continuous change. Our study shows a continuous change in PHC organizational routines aimed at improving the quality of care for patients.

Organizational factors unintentionally affected all three organizational routines in our results. Our study was conducted in a specific organizational context in Sweden where PHC units are rather autonomous from one another. Previous studies found structural and local factors, such as the absence of coordination and regulation, insufficient preparedness and capacity for change decreased motivation in healthcare providers [17, 18]. While PHC units were not included in the planning and implementation of CPPs, our results suggest that they were well-prepared for the change that this involved and continued to be highly motivated. Instead, the organizational factors that unintentionally affected the organizational routines were related to the strong division between nurses and physicians, as well as between primary and secondary care. In other words, nurses were not involved in the adoption process of CPPs and PHC units were not asked and thereby not able to give feedback on shared guidelines between care levels. Additionally, such stiffness to change working processes within healthcare organizations may even strengthen the fragmentation of care and old hierarchical positioning between primary and secondary care levels, even though CPPs intend to simplify the diagnosis process for patients. Thus, conditions of the organizational environment as well as in the organization itself shaped the adoption of this new tool and hence had unintended consequences.

### Strengths and limitations of the research design

The use of Grounded Theory method was a valuable strategy to explore openly and flexibly the research aim. Additionally, the multidisciplinary research team (nursing, social and medical sciences with a gender-equal group) brought together expertise from PHC and cancer research to contribute different perspectives during data collection and analysis. Northern Sweden is a challenging setting for the provision of PHC because the population is highly dispersed in a large geographical area, with only a few large cities. We included a variety of PHC units to reflect this setting: small and large-sized units in both urban and rural areas. In addition, we collected data among small and big groups with professions participating together or separated. However, in data collection, we had to account for PHC units daily work and their difficulty allocating time, with limitations to 45–60 min for group interviews and some could not participate because of it. Therefore, two groups were small with two participants. Variation in groups nonetheless provided opportunities for participants to talk more in-depth as well as stimulate discussions within and between profession groups. Moreover, the interviewers had complemented inside and outside perspectives to collect as rich and diverse data as possible. Both perspectives were essential for us to actively handle our preconceptions and thereby ground emerging categories in participants’ actions and meanings.

## Conclusion

CPPs is a standardized tool implemented by the healthcare systems of several countries to improve and speed up the diagnosis and treatment of cancer. In Sweden, PHC units have a central role in cancer detection and treatment. Therefore, we focused here on how these units had adjusted organizational routines to utilizing CPPs and the intended and unintended consequences this has had. Our study illustrates that the internal know-how within PHC needs to be included and integrated into the healthcare system to facilitate and improve the use of new tools such as CPPs. Further, it shows that the relationships between different levels of care within the healthcare systems, and the degree of division or coordination between them should be taken into account when developing and introducing new tools such as CPPs. In the future, further research is needed on the unintended consequences of the adoption of innovations for health care organizations in diverse contexts internationally in order to generate a better knowledge based. Our results contribute to this emerging knowledge base through its focus on the adoption of new CPPs in the context of PHC. This contribution is crucial because standardized patient pathways are not limited to cancer care but also are being introduced in other disease-specific processes in healthcare systems.

## Data Availability

The datasets generated and/or analysed during the current study are not publicly available due to the data includes information that could compromise research participants’ privacy and consent but are available from the corresponding author on reasonable request.

## Abbreviations

CPP: Cancer Patient Pathways
PHC: Primary HealthCare
